# Supplementary material for: Recognition of flight cadets brain functional magnetic resonance imaging data based on machine learning analysis
Source: PLoS One. 2025 Jun 5;20(6):e0324081. doi: 10.1371/journal.pone.0324081 (PMC12140279; doi:10.1371/journal.pone.0324081)
Supplement: Attachment — Submitted filename: Response to Reviewers.pdf [file pone.0324081.s003.pdf]

I Original comments of Reviewer #1:

Overall, this is a very interesting manuscript that I would suggest is published with minor edits. The manuscript is well written with clear explanation and logic. I suggest minor edits for consideration below.

Response to Reviewer #1:

Dear Reviewer,

We are truly grateful to your positive comments and thoughtful suggestions on our manuscript (Manuscript Number: PONE-D-24-48304 entitled "Recognition of flight cadets brain functional magnetic resonance imaging data based on machine learning analysis"). Based on these comments and suggestions, we have made careful modifications on the original manuscript. All changes made to the revision are in light blue, and all changes used to revision in response letter marked with "****". We hope the new manuscript will meet with your approval. Below you will find our point-by-point responses to your comments/ questions.

1) **Text of reviewer's comment:** 1. Please provide an explanation on the exposure of the control group. Was this group only involved in ground school training without any flight experience? Are these trainees in another carrier field? This will help the reader understand what is meant by 'trainees from the ground program.' I see that 0 hours of flight training was recorded in Table 2.

Response: Thank you very much for your comments. We will provide a more detailed description of the background of the control group in the "Participants" section to help readers better understand the differences between the two groups.

Revision (lines 156-162):

"The control group consisted of cadets who received no flight training and only participated in ground-based theoretical courses (such as aviation theory, meteorology, and navigation principles). All ground-based cadets came from the engineering discipline of the same institution, and possessed neither a flight license nor any

experience in flight simulation. Their training content significantly differed from that of the flight cadets, focusing primarily on theoretical learning and ground operation skills, rather than practical flight tasks."

2) **Text of reviewer's comment:** 2. Table 2 – I am not clear what 39/0 and 37/0 mean in the Sex category. I would suggest reporting the n instead of the %, or put the % within parentheses.

Response: We greatly appreciate your identification of the issue. In accordance with your suggestion, we have revised the data presentation in the gender column of Table 2 to: Gender (% male), and the specific values for both the flight cadets and the control group in this column have been updated to 100%.

3) **Text of reviewer's comment:** 3. On line 271, I might suggest adding "where feature is defined as…" to add clarity for the reader.

Response: We sincerely appreciate your suggestion. We will include an additional explanation of the term "feature" in the revised manuscript to provide readers with a clearer understanding of this concept.

Revision (lines 323-324):

"In this study, features are defined as quantitative metrics extracted from resting-state fMRI data, including BOLD signals, FC, ALFF, and ReHo."

4) **Text of reviewer's comment:** 4. Line 295 – I would suggest changing the word 'hue' to 'color.'

Response: Thank you very much for your suggestion. In the revised manuscript, we have changed "hue" to "color."

5) **Text of reviewer's comment:** 5. I would suggest Figure 7 and 8 (with explanation) be within the results section with interpretation in the discussion section.

Response: Thank you very much for your suggestion. Regarding your suggestion to move Figure 7 and Figure 8, along with their explanations, to the Results section and

then interpret them in the Discussion section, we understand that you aim to make the article's structure clearer and its logic more coherent through this adjustment. However, in our current article framework, these two figures serve as visual representations of the key findings in the research results. Their main purpose is to display the key brain regions and connections identified based on different indicators (BOLD signals and FC). In the Results section, we primarily focus on presenting the classification performance at the data level (such as AUC, sensitivity/specificity) and the screening results of key features (Table 3, Figure 1 - 4). In the Discussion section, we conduct in - depth discussions from perspectives such as neuroscience theories and the actual impact of flight training, in combination with these findings. Placing Figure 7 and Figure 8 in the Discussion section can more effectively help readers visually observe the positions and distributions of the relevant brain regions and connections while understanding the association between theory and practice, thus enabling them to better comprehend our research conclusions. Therefore, considering the integrity and logic of the article, we hope to retain the current positions of the figures.

6) **Text of reviewer's comment:** 6. The explanation of the BOLD signal was informative for the rest of the manuscript. I might recommend the authors provide a similar concise explanation of how the reader should conceptualize FC, ALFF, and ReHo as there is no explanations of what these functions are.

Response: Thank you very much for your suggestion. In our manuscript, we indeed did not provide detailed explanations for these indicators, which may cause inconvenience to readers. We will briefly introduce FC, ALFF, and ReHo in the "Feature Extraction" section. We will illustrate their roles in brain function research and explain how to calculate these indicators from fMRI data.

Revision (lines 220-230, lines 212-215):

"In this study, the calculation of ALFF involves performing a Fourier transform on the time series of each voxel to obtain the power spectrum within the low-frequency range (0.01 - 0.08 Hz). The square root of this power spectrum is then computed to derive the ALFF value for each voxel. This value effectively reflects the intensity of spontaneous

neural activity in the brain and provides a critical metric for assessing the brain's intrinsic functional state. ReHo, on the other hand, is computed using Kendall's coefficient of concordance (KCC). Specifically, the KCC value is calculated by comparing the time series of each voxel with those of its neighboring voxels, which measures the temporal coherence within local brain regions. This metric is crucial for understanding the synchrony of neural activity within localized brain areas, contributing to the exploration of the characteristics of local brain functional integration."

"FC can reflect the degree of connection tightness between various brain regions in the brain functional network, which helps to understand how different regions of the brain cooperate with each other functionally."

7) **Text of reviewer's comment:** 7. Lines 552 – 557: Other limitations – only trainee pilots in one country with limited flying experience. I would suggest commenting on gender and handedness (see comment about Table 2). Additionally, each ML model had <100% sensitivity and specificity. I would suggest adding a line discussing how there are factors not identified in these models, which suggest they are incomplete.

Response: Thank you very much for your comments. We will supplement the description of limitations and comments on gender and handedness in the revised manuscript according to your suggestions. Regarding the issue you raised that the sensitivity and specificity of each machine - learning model are less than 100%, we are well aware of your concern about the accuracy and integrity of the models. However, in the field of machine learning, due to various factors such as data complexity and the limitations of the models themselves, especially considering that fMRI data is more difficult to reach a high level due to individual differences, measurement noise, and environmental factors. In all the studies related to fMRI combined with machine learning referenced in this research, the evaluation metrics for the machine learning models did not reach 100%. From this perspective, the failure of the model performance to reach 100% is a normal phenomenon in this field and is not a problem unique to this study.

Furthermore, the core goal of the machine - learning field is to discover potential patterns through data - driven methods rather than to pursue absolute perfect performance. In fact, no ML model can completely avoid misclassification, especially when dealing with complex fMRI data. Therefore, we believe that the failure of the model's sensitivity and specificity to reach 100% does not mean that the model is "incomplete", but rather reflects the limitations and practicality of machine learning in real - world applications.

Revision (lines 604-607):

“Additionally, all participants were from the CAFUC and were limited to male right - handed cadets. Although this approach reduced the impact of potential confounding factors, the samples were sourced from the same region. Future research could consider incorporating more diverse samples.”

8) **Text of reviewer’s comment:** 8. I am not clear how any conclusions can be drawn from this project about how this ML model could be used to aid pilot selection. This study did not aim to identify who did/did not pass flight training. Instead, this study aimed to identify whether ML models can identify whether someone did or did not complete a certain degree of flight training. I would suggest removing this line. A line could be added about how these data could inform future working aiming to predict pilot performance in flight training/selection.

Response: Thank you very much for your comments. Indeed, the primary objective of this study is not to directly determine who can pass flight training, but rather to explore whether the machine learning model can differentiate between cadets who have undergone flight training and those who have not. We will remove the direct conclusion in the manuscript suggesting that the model could assist in pilot selection. Meanwhile, as per your suggestion, we will add content to explain that these data can provide references for future research on guiding pilot flight training.

Revision (lines 592-601):

“The key brain regions identified in this study, along with the demonstrated

effectiveness of the ML model in differentiating individuals who have received flight training, offer highly valuable reference for subsequent research. These research findings are expected to provide direction for constructing more accurate pilot prediction models. Furthermore, conducting an in - depth analysis of the brain networks where the key brain regions are located can help scientifically guide the curriculum arrangement of flight training and rationally plan the key content of training subjects according to the functional characteristics of each brain region. In this way, flight training can better conform to the development laws of brain functions, improve training effectiveness, and provide strong support for cultivating high - quality pilots."

9) **Text of reviewer's comment:** 9. Line 569, I would recommend changing the word 'indicating' to 'suggesting'.

Response: Thank you very much for your suggestion. We have changed "indicating" to "suggesting" in the revised manuscript.

10) **Text of reviewer's comment:** 10. Line 571, I would suggest another term besides 'regular' trainees.

Response: Thank you very much for your suggestion. we have changed "regular trainees" to "non - flight trainees" in the revised manuscript.

11) **Text of reviewer's comment:** 11. Line 572-576, again, I am not sure how any conclusions can be drawn about 'pilot screening and training optimization.' I would suggest removing this line. This study demonstrated that a ML model can identify those who did and did not complete flight training and provides insight on neural connectivity. It would be reasonable suggest a line how future research could employ these data to support those objectives.

Response: Thank you very much for your comments. Regarding how the conclusion of pilot training optimization was reached, we elaborated on it in response 8).

12) **Text of reviewer's comment:** 12. A line will need to be added stating whether the

authors will provide their primary data upon request.

Response: Thank you very much for your suggestion. We have submitted the data availability statement through the paper's backend system, so we didn't add an additional statement in the paper itself.

II Original comments of Reviewer #2:

The manuscript submitted by Le et al. examines the neural profile of flight cadets and how it differs from ground program cadets. All participants underwent a resting state fMRI scan which was used by the authors to extract four different metrics of brain activity (BOLD signal, functional connectivity, amplitude of low frequency fluctuation, and regional homogeneity). The authors developed five different classification models using machine learning to determine the best model(s) to analyze the resting state fMRI data. Overall, the BOLD signal was the best metric to classify flight cadets while the logistic regression and Gaussian Naïve Bayes models exhibited higher classification accuracy relative to other model types. Moreover, specific brain regions in the default mode, frontoparietal, somatomotor, and visual networks possess different connectivity patterns in flight cadets. In general, the manuscript examines an interesting topic that may guide future studies in personnel selection for certain occupations. However, there are a few points that the authors should address in the manuscript before acceptance can be considered.

Response to Reviewer #2:

Dear Reviewer,

We are truly grateful to your positive comments and thoughtful suggestions on our manuscript (Manuscript Number: PONE-D-24-48304 entitled "Recognition of flight cadets brain functional magnetic resonance imaging data based on machine learning analysis"). Based on these comments and suggestions, we have made careful modifications on the original manuscript. All changes made to the revision are in light blue, and all changes used to revision in response letter marked with "****". We hope the new manuscript will meet with your approval. Below you will find our point-by-point responses to your comments/ questions.

1) **Text of reviewer's comment:** Major 1. Introduction (page 4, lines 67-68; page 5, lines 98-99): There are many terms that the authors mention but do not define or describe. Terms such as SN, DMN, CEN, ALFF, ReHo are all technical terminology that brain imaging researchers would understand, but for the average reader, which

would likely be the case for a PLoS One audience, these terms should be defined. Some of these terms, such as DMN, are not defined until the Discussion (page 25, lines 434-439) when it would be more appropriate to define them in the Introduction instead. The aforementioned terms only require 1-2 sentences each to describe in the Introduction while a more detailed description of the relevant terms, such as DMN, can be elaborated on in the Discussion.

Response: Thank you very much for your comments. We will add brief definitions of the relevant terms in the introduction section to provide a better reading experience for PLoS One readers. The meanings of the indicators (ReHo, ALFF) will be explained in the "Feature Extraction" section, with specific revisions provided in response to Major 5.

Revision (lines 64-77):

"In brain network research, multiple networks are typically divided based on functional connectivity and anatomical structures, such as the Salience Network (SN), Default Mode Network (DMN), Central Executive Network (CEN), etc. Each network is responsible for specific cognitive and behavioral functions. The SN is mainly responsible for identifying and integrating sensory information, guiding attention and behavior. The DMN is most active when the brain is in a resting state and is involved in internal thought processes such as self - reflection, memory recall, and future planning. The CEN is associated with cognitive control and attention and is responsible for performing complex tasks, such as working memory, decision - making, and problem - solving. Previous studies have found that in terms of these brain networks, pilots exhibit enhanced functional connectivity between the SN, DMN, and CEN, while at the same time, the connectivity within each of these three networks decreases, and the temporal properties of functional dynamics increase."

2) **Text of reviewer's comment:** Major 2. Introduction (page 5, lines 95-97): The study rationale seems incomplete. The authors do not elaborate why it is important to study brain function in healthy individuals. For example, an explanation of what constitutes flight training and how it is unique from other occupations (e.g., ground

cadets) would be helpful. Similarly, the authors mention drug/alcohol use (lines 106-109) but it is unclear how this even relates to the study question.

Response: Thank you very much for your comments. In our research, the main purpose of referring to the healthy population was to emphasize that, compared with patient groups, the changes in brain functions between flight cadets and ground cadets might be more subtle and less noticeable. This further highlights the rationality and superiority of the machine - learning approach in dealing with such fMRI data, as well as the innovation of this study. Certainly, we fully agree with the reviewer's suggestions and will supplement the following content in the Introduction section: First, clearly expound on the significance of studying the brain functions of flight cadets; second, explain in detail the differences between flight training and ground - trainee training to enhance the logic and rationality of the research. In our study, the mention of smoking and alcoholism was to illustrate that BOLD signals can reflect brain changes, thus can be used as machine - learning indicators and for discussions on physiological significance. Considering that such an expression may lead to ambiguity, we have decided to delete this statement.

Revision (lines 104-118):

"In this study, all the research subjects were from the healthy population. However, the flight training that flight cadets participate in encompasses elements such as high - pressure decision - making, multi - sensory integration (including aspects like vision, hearing, and spatial perception), and rapid cognitive switching, which are significantly different from the theoretical or operational tasks that ground cadets engage in. For example, in the aircraft cockpit, flight cadets must process a vast amount of information in real - time, such as instrument panel data, tower signals, and radio communication content, and make quick decisions. This intense cognitive training is highly likely to have a unique impact on brain functions. Therefore, exploring the changes in the brain functions of healthy flight cadets can lay the foundation for a deeper understanding of the mechanisms by which this special vocational training shapes the brain, and also contribute to the development of more efficient training strategies. Given that the changes in brain functions among healthy individuals are often more subtle and difficult

to detect, this study employs the ML method to conduct an in - depth investigation of the fMRI data of flight cadets' brains."

3) **Text of reviewer's comment:** Major 3. Introduction (page 6, lines 110 – 119): This seems like an exploratory study, which should be made clear in the introduction. Furthermore, what were the authors' hypotheses for the study questions/objectives?

Response: Thank you very much for your comments. This study indeed possesses an exploratory nature, which will be explicitly highlighted in the introduction. Regarding the research hypothesis, based on the relevant studies discussed in the introduction, we hypothesize that flight training will induce changes and effects on cognitive abilities, and that machine learning methods can effectively capture the differences in various brain fMRI metrics between flight cadets and ground cadets, thus enabling precise classification.

Revision (lines 129-134):

"This study is of an exploratory nature. Integrating previous relevant research, we hypothesize that flight training will bring about changes and exert impacts on the brain's cognitive abilities. Moreover, we assume that machine - learning methods can capture the differences in various brain fMRI indices between flight cadets and ground cadets, thus enabling accurate classification."

4) **Text of reviewer's comment:** Major 4. Methods (page 8, 163-165): The authors removed the signal from cerebrospinal fluid, white matter, and head motion. It is common procedure for the resting state fMRI data to also remove physiological signals, such as heart beat and respiration. Could the authors explain why this was not collected and/or regressed out and how this would impact their resting state signal metrics?

Response: Thank you very much for your comments. In the data pre - processing stage of this study, we fully considered the potential impact of physiological signals such as respiration and heartbeat on the experimental results. Therefore, in the seventh step of pre - processing, a filtering operation was carried out to limit the signal frequency within the range of 0.01 - 0.08Hz, removing signals outside this range. From a physiological perspective, the heart rate of healthy adults generally ranges from 60 to

100 beats per minute. When converted, the corresponding frequency range is approximately 1Hz - 1.67Hz. The respiratory rate usually ranges from 12 to 20 breaths per minute, with a corresponding frequency range of approximately 0.2Hz - 0.33Hz. In the selection of subjects for this study, through strict screening, we ensured that all the basic physiological indicators of the subjects included in the study were within the healthy standard range. Based on the above premises, the filtering method adopted in this study can theoretically maximize the suppression of noise interference introduced by physiological signals such as respiration and heartbeat in the data, thereby significantly improving the quality and reliability of the data. In addition, in research in related fields, many scholars in the past commonly used filtering methods during the pre - processing stage to reduce the impact of physiological signals such as heartbeat and respiration when dealing with fMRI data. These scholars conducted in - depth research on the fMRI indicators obtained after filtering and successfully obtained results with statistical significance and scientific value[1-3]. These research findings have confirmed the effectiveness and feasibility of filtering in removing the noise of physiological signals.

5) **Text of reviewer's comment:** Major 5. Methods (Feature Extraction, page 8): The authors only explained how they calculated BOLD signal and FC. Where is the description for how ALFF and ReHo were computed?

Response: Thank you very much for your comments. In the revised manuscript, we will elaborate on the descriptions of the calculation methods for ALFF and ReHo in the "Feature Extraction" section.

Revision (lines 220-230):

"In this study, the calculation of ALFF involves performing a Fourier transform on the time series of each voxel to obtain the power spectrum within the low-frequency range (0.01 - 0.08 Hz). The square root of this power spectrum is then computed to derive the ALFF value for each voxel. This value effectively reflects the intensity of spontaneous neural activity in the brain and provides a critical metric for assessing the brain's intrinsic functional state. ReHo, on the other hand, is computed using Kendall's

coefficient of concordance (KCC). Specifically, the KCC value is calculated by comparing the time series of each voxel with those of its neighboring voxels, which measures the temporal coherence within local brain regions. This metric is crucial for understanding the synchrony of neural activity within localized brain areas, contributing to the exploration of the characteristics of local brain functional integration."

6) **Text of reviewer's comment:** Major 6. Methods (Feature extraction, pages 8-10): Have the authors considered using graph theory as another resting state fMRI metric to be compared in the study? Graph theory allows resting state fMRI data to be analyzed with more granularity which is particularly relevant in this study when various metrics are being compared head-to-head [see Medaglia (2017) Neuroimaging Clin N Am, 27(4):593-607; Lv et al., (2018) AJNR Am J Neuroradiol, 39(8):1390-1399]. Given that the authors already have 116 brain regions outlined in Table 1 that they used in calculating the BOLD signal and FC, they can perform graph theory on these same regions to extract values such as clustering, path length, or efficiency.

Response: Thank you very much for your invaluable suggestion regarding the inclusion of graph theory as a comparative indicator for analyzing resting-state fMRI data. Graph Theory, as a powerful tool for analyzing resting - state fMRI data, can provide more fine - grained network topological information through indices such as clustering coefficient, path length, and efficiency. However, in the current study, after careful consideration, we have decided not to include graph - theory - related indices for the time being. The indices we have chosen, namely FC, BOLD, ALFF, and ReHo, possess unique advantages. BOLD can reflect changes in the activation level of brain regions. FC can directly reflect the coordination of functional activities between different brain regions and is excellent in studying the interactions within the brain network. ALFF focuses on the amplitude changes of spontaneous neural activities in the brain, which helps us gain in - depth insights into the alterations in the intensity of neural activities in specific brain regions. ReHo, on the other hand, concentrates on the synchrony of neural activities in local brain regions and can effectively capture the characteristics of

local functional integration in the brain. These indices have been widely used in numerous previous studies of the same kind and have been proven to be highly targeted and explanatory for the flight - trainee group we are researching[4, 5]. They can directly reflect the changes in the brain functions of flight cadets.

Nonetheless, the reviewer's suggestion is highly inspiring. In subsequent research, we will give full consideration to incorporating graph - theoretic indices to further deepen and broaden the scope of our study. Once again, thank you for your advice, which provides important reference for our future research.

7) **Text of reviewer's comment:** Major 7. Methods (page 9, lines 183-186) and Results (page 15, Table 3): It appears that the resting state fMRI metrics with the most features that have been extracted (29,000 for BOLD signal and 6,670 for FC) and selected (40 for BOLD signal and 141 for RC) are ultimately the features that will perform the best in the ML algorithms. While I recognize the authors applied more stringent p-value thresholds for the BOLD and RC metrics, the ALFF and ReHo metrics appear to already be at a disadvantage from the beginning as fewer features were extracted to be used in the ML model comparison and evaluation. In other words, how would the results differ if all 4 resting state fMRI metrics used the same number of features (e.g., only the top 10 features for each metric) for ML model comparison and evaluation?

Response: Thank you very much for your comments. We understand the reviewer' s concern regarding the differences in the number of features. However, the disparity in feature count does not affect the study's results for the following reasons:

1. Under more stringent feature selection criteria, the BOLD and FC indicators retain a relatively larger number of features, which highlights the inherent superiority of BOLD and FC signals themselves. This is because more comprehensive and distinctive metrics tend to contain more valuable information, which is why a greater number of features are retained during the feature selection process. Moreover, past studies combining ML methods with fMRI metrics have not standardized the number of features for each metric post-feature selection[6].

2. In supplementary analysis, we limited the number of features for each metric fed into the ML model to 10. The results still showed that BOLD and FC indicators performed the best. In this supplementary analysis, we applied standardized feature selection to each metric, using the top 10 features for model comparison. To simplify the analysis process and avoid redundant model evaluations, we selected Gaussian Naive Bayes (GNB) as the base classifier, which demonstrated the best performance across all metrics. We presented the results using ROC curves. As shown in Figure 1, BOLD signals performed the best, followed by FC, while ALFF and ReHo showed the weakest performance. These results clearly indicate that the classification performance based on these metrics is not solely determined by the number of features. Instead, it is influenced by various factors, including the inherent characteristics of the metrics, their ability to capture relevant information, and the interactions between the features and the classification model.

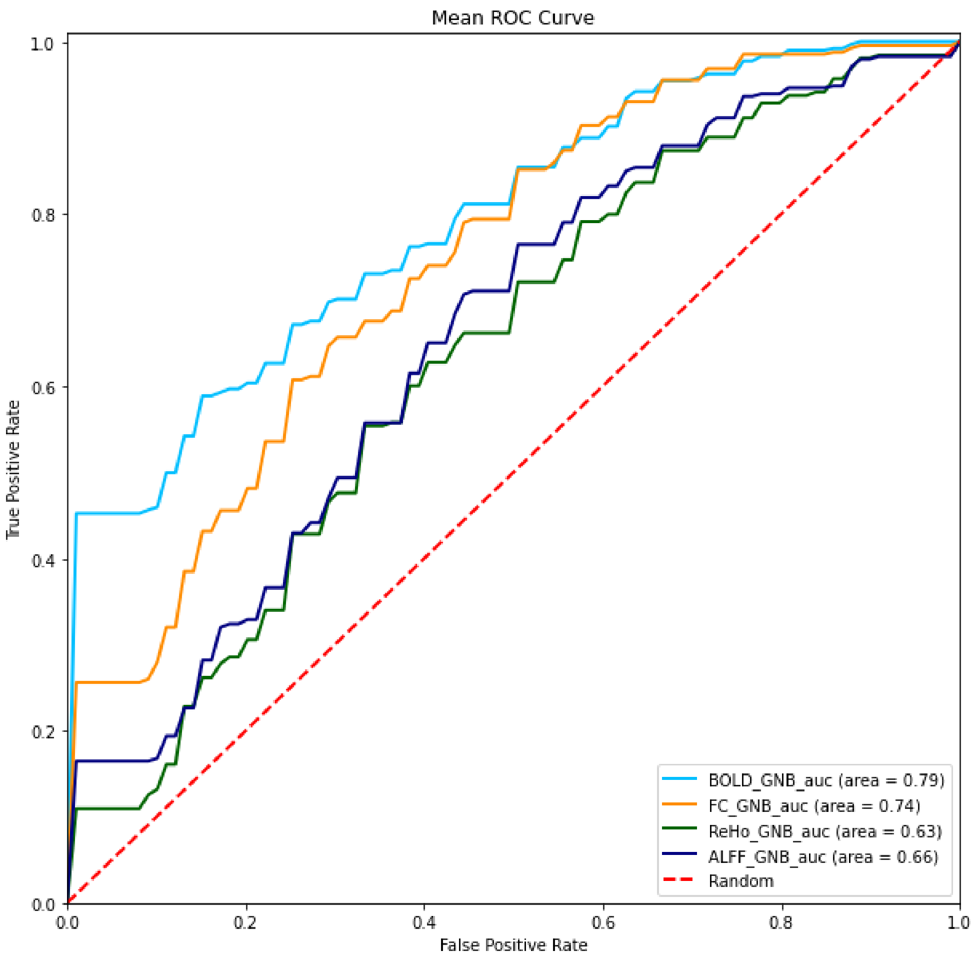

Fig 1. Comparison of ROC for Four Metrics with the Number of Features Limited to 10

8) **Text of reviewer's comment:** Major 8. Methods (pages 12-13, lines 221-223): The choice of the 5 classification models (GNB, SVM, RF, LR, and XGBoost) appears arbitrary. Please explain the rationale for these 5 model types over other machine learning models. Would the authors obtain similar results if they used k-nearest neighbor (KNN) or principal component analysis (PCA), as an example?

Response: Thank you very much for your comments. We will include the reasons for choosing these five models in the "ML model establishment" section of the revised manuscript. Our selection of the five supervised learning models, namely GNB, SVM, RF, LR, and XGBoost, is by no means arbitrary. Instead, it is founded on the following scientific justifications and research objectives:

1. Rationality of Model Selection

Coverage of Algorithmic Diversity: The models selected span probability models (GNB), linear models (LR), kernel methods (SVM), and ensemble learning (RF, XGBoost). This enables a comprehensive evaluation of the performance disparities among different types of algorithms when applied to the same dataset.

Applicability in Neuroimaging: The five categories of models we chose are widely utilized in fMRI classification tasks and are characterized by high accuracy and robustness[7-11]. For example, RF, SVM, and XGBoost perform outstandingly when handling complex data[2, 6, 12, 13]. LR, being a classic linear classification model, is simple, easily comprehensible, and highly interpretable. GNB, based on Bayes' theorem, features high computational efficiency and can rapidly process large - scale data, especially in data scenarios where features are relatively independent[11, 14].

2. Comparison with Other Models:

KNN: While KNN is an effective classification method, it is highly sensitive to data distribution and noise, and its computational complexity is relatively high. In the context of our study, KNN may not offer the same level of robustness as SVM or GNB.

PCA: PCA is an unsupervised dimensionality reduction technique, rather than a classification model. While it can be used for feature extraction and data preprocessing, it is not directly applicable to classification tasks[7, 15]. In the early stages of this study,

we attempted to utilize PCA for feature dimensionality reduction with the aim of feature selection. However, the results did not meet expectations. As the study progressed, further analysis led us to hypothesize that the suboptimal performance of PCA may stem from its unsupervised nature. This method does not account for the class label information associated with the data when performing dimensionality reduction. In practical scenarios, when the class information within the data is crucial to the reduction process, PCA's inability to capture this essential information hampers its ability to achieve the desired dimensionality reduction, thereby affecting the results of feature selection.

In conclusion, we selected these five models to ensure diversity and complementarity while considering the research objectives and data characteristics. These models have demonstrated strong adaptability and robustness across various datasets and tasks, providing a comprehensive evaluation for our study. Although KNN and PCA may have their advantages in certain situations, they are not fully suitable for the current research task.

Revision (lines 270-277):

"The five categories of models we chose are widely utilized in fMRI classification tasks and are characterized by high accuracy and robustness. For example, RF, SVM, and XGBoost perform outstandingly when handling complex data. LR, being a classic linear classification model, is simple, easily comprehensible, and highly interpretable. GNB, based on Bayes' theorem, features high computational efficiency and can rapidly process large - scale data, especially in data scenarios where features are relatively independent."

9) **Text of reviewer's comment:** Major 9. Discussion (pages 24-25, lines 416-425): The authors discuss the results from the perspective of neural changes due to cadet training. While this is a plausible explanation, this seems a bit misleading for this study since the authors did not compare flight cadets before and after a training course nor did they compare separate groups of flight cadets (experienced vs. novice). Instead, the authors compared a group of flight cadets versus ground cadets, the latter being a different occupation altogether. One cannot rule out the possibility that the neural

profile for flight cadets is already different from ground cadets at the outset and may (or may not) be related to performance/success in flight cadet training, which of course requires further research to investigate.

Response: Thank you very much for your comments. In this study, both the experimental group and the control group were composed of cadets from the same enrollment batch. These cadets had similar entrance examination scores. Throughout their undergraduate studies, the learning content, total credit hours, and theoretical class hours of the two groups of cadets were essentially equivalent. In the initial selection of flight cadets, the emphasis was on physical fitness and visual acuity, and the selection requirements in other aspects were the same as those for ground cadets. Therefore, from the beginning, there were no significant differences in neural characteristics between the two groups of cadets. However, flight cadets had to undergo professional flight training during their undergraduate years, including simulation training and actual aircraft operation, and ultimately obtain private and commercial pilot licenses issued by the Civil Aviation Administration of China. Thus, the main difference between these two types of cadets lies in the impact of flight training. There is substantial evidence supporting the influence of flight training on the brains of pilots, and relevant literature has also been cited in this manuscript. For example, the study by Chen et al. demonstrated a significant association between flight training and white - matter fiber tracts, degree centrality, and functional connectivity within the default mode network. Additionally, as mentioned in the Introduction, Adamson, Xu et al. also confirmed the effects of flight training on brain structure and function. These findings provide important support for the arguments presented in this manuscript.

Revision (lines 150-156):

"In this study, both the experimental group and the control group were composed of cadets from the same enrollment batch. These cadets had similar entrance examination scores. Throughout their undergraduate studies, the learning content, total credit hours, and theoretical class hours of the two groups of cadets were essentially equivalent. In the initial selection of flight cadets, the emphasis was on physical fitness and visual acuity, and the selection requirements in other aspects were the same as those for ground

cadets.”

10) **Text of reviewer’s comment:** Minor 1. Introduction, page 5, line 106: Please add the word “functional” in front of magnetic resonance imaging.

Response: Thank you very much for your comment. In the revised manuscript, we have added "functional" at that position.

11) **Text of reviewer’s comment:** Minor 2. Methods, page 7 (line 146) and page 8 (line 152): Please explicitly state the voxel size for the T1 and resting state fMRI scans. Are these 4 × 4 × 4 mm voxels?

Response: Thank you very much for your comment. We have added the voxel sizes for T1 and fMRI scans in the revised manuscript. T1: 1 × 1 × 1 mm voxels; fMRI: 3 × 3 × 3 mm voxels.

12) **Text of reviewer’s comment:** Minor 3. Results, page 14, Table 2: Please clarify the number of participants in the flight cadet group. Table 2 says N = 26, but on page 14, line 243 it indicates that N = 39.

Response: Thank you very much for your comment. We have corrected the number of flight cadets in Table 2 to 39 (N = 39) in the revised manuscript.

13) **Text of reviewer’s comment:** Minor 4. Discussion, page 26-27, lines 458-462: Both sentences seem redundant, please consider rewording or removing one of these sentences.

Response: Thank you very much for your comment. We have deleted the second repeated sentence in the revised manuscript.

14) **Text of reviewer’s comment:** Minor 5. Please check for minor spelling typos throughout the manuscript (e.g., page 5, line 98 reads as: “… resting-state brain function, including Amplitude of ALFF, functional connectivity …” but either the words “Amplitude of” is not required or the letters “FF” should be spelled out).

Response: Thank you very much for your comment. We have checked and corrected

the spelling throughout the revised manuscript.

1. Xu S, Zhang Z, Li L, Zhou Y, Lin D, Zhang M, et al. Functional connectivity profiles of the default mode and visual networks reflect temporal accumulative effects of sustained naturalistic emotional experience. NeuroImage. 2023;269:119941.
2. Sun Y, Zhao L, Lan Z, Jia X-Z, Xue S-W. Differentiating boys with ADHD from those with typical development based on whole-brain functional connections using a machine learning approach. Neuropsychiatric disease and treatment. 2020:691-702.
3. Chen X, Wang Z, Jiang H, Meng Y, Wang H, Li Y, et al. Flight training changes the brain functional pattern in cadets. Frontiers in Neuroscience. 2023;17:1120628.
4. Chen X, Wang Q, Luo C, Yang Y, Jiang H, Guo X, et al. Increased functional dynamics in civil aviation pilots: evidence from a neuroimaging study. Plos one. 2020;15(6):e0234790.
5. Xu K, Liu R, Chen X, Chen X, Yang Y, Wang Q, et al. Research on brain functions related to visual information processing and body coordination function of pilots based on the low-frequency amplitude method. Frontiers in Human Neuroscience. 2023;17:796526.
6. Yan B, Xu X, Liu M, Zheng K, Liu J, Li J, et al. Quantitative identification of major depression based on resting-state dynamic functional connectivity: a machine learning approach. Frontiers in neuroscience. 2020;14:500338.
7. Khosla M, Jamison K, Ngo GH, Kuceyeski A, Sabuncu MR. Machine learning in resting-state fMRI analysis. Magnetic resonance imaging. 2019;64:101-21.
8. Dadi K, Rahim M, Abraham A, Chyzhyk D, Milham M, Thirion B, et al. Benchmarking functional connectome-based predictive models for resting-state fMRI. NeuroImage. 2019;192:115-34.
9. Wu X, Zhai F, Chang A, Wei J, Guo Y, Zhang J. Application of machine learning algorithms to predict osteoporosis in postmenopausal women with type 2 diabetes mellitus. Journal of endocrinological investigation. 2023;46(12):2535-46.
10. Yadav R, Gautam A, Mishra RB. Classification of alzheimer using fmri data and brain network. Comput Sci Inf Technol. 2018;2018:109-19.
11. Saccà V, Sarica A, Novellino F, Barone S, Tallarico T, Filippelli E, et al. Evaluation of machine learning algorithms performance for the prediction of early multiple sclerosis from resting-state FMRI connectivity data. Brain imaging and behavior. 2019;13:1103-14.
12. Wee C-Y, Yap P-T, Zhang D, Denny K, Browndyke JN, Potter GG, et al. Identification of MCI individuals using structural and functional connectivity networks. Neuroimage. 2012;59(3):2045-56.
13. Curtin P, Neufeld J, Curtin A, Arora M, Bölte S. Altered periodic dynamics in the default mode network in autism and attention-deficit/hyperactivity disorder. Biological psychiatry. 2022;91(11):956-66.
14. Pereira F, Mitchell T, Botvinick M. Machine learning classifiers and fMRI: a tutorial overview. Neuroimage. 2009;45(1):S199-S209.
15. Avanzo M, Wei L, Stancanello J, Vallieres M, Rao A, Morin O, et al. Machine and deep learning methods for radiomics. Medical physics. 2020;47(5):e185-e202.
