# Supplementary material for: Recognition of flight cadets brain functional magnetic resonance imaging data based on machine learning analysis
Source: PLoS One. 2025 Jun 5;20(6):e0324081. doi: 10.1371/journal.pone.0324081 (PMC12140279; doi:10.1371/journal.pone.0324081)
Supplement: Attachment — Submitted filename: Review Comments.docx [file pone.0324081.s001.docx]

Overall, this is a very interesting manuscript that I would suggest is published with minor edits. The manuscript is well written with clear explanation and logic. I suggest minor edits for consideration below.

To the Editor:

I would suggest a neuroradiologist or a MR physicist reviews the data acquisition and pre-processing section.

I would suggest a biostatistician and/or a machine learning scientist is consulted to review the tools and analysis of this study. While I have no overt concerns, I am not fully qualitied to review the ML methodologies employed.

Introduction

The explanation of the BOLD signal was informative for the rest of the manuscript. I might recommend the authors provide a similar concise explanation of how the reader should conceptualize FC, ALFF, and ReHo as there is no explanations of what these functions are.

Materials and Methods

Please provide an explanation on the exposure of the control group. Was this group only involved in ground school training without any flight experience? Are these trainees in another carrier field? This will help the reader understand what is meant by ‘trainees from the ground program.’ I see that 0 hours of flight training was recorded in Table 2.

Table 2 – I am not clear what 39/0 and 37/0 mean in the Sex category. I would suggest reporting the n instead of the %, or put the % within parentheses.

On line 271, I might suggest adding “where feature is defined as…” to add clarity for the reader.

Line 295 – I would suggest changing the word ‘hue’ to ‘color.’

I would suggest Figure 7 and 8 (with explanation) be within the results section with interpretation in the discussion section.

Lines 552 – 557:

Other limitations – only trainee pilots in one country with limited flying experience. I would suggest commenting on gender and handedness (see comment about Table 2). Additionally, each ML model had <100% sensitivity and specificity. I would suggest adding a line discussing how there are factors not identified in these models, which suggest they are incomplete.

I am not clear how any conclusions can be drawn from this project about how this ML model could be used to aid pilot selection. This study did not aim to identify who did/did not pass flight training. Instead, this study aimed to identify whether ML models can identify whether someone did or did not complete a certain degree of flight training. I would suggest removing this line. A line could be added about how these data could inform future working aiming to predict pilot performance in flight training/selection.

Line 569, I would recommend changing the word ‘indicating’ to ‘suggesting’

Line 571 – I would suggest another term besides ‘regular’ trainees

Line 572-576 – again, I am not sure how any conclusions can be drawn about ‘pilot screening and training optimization.’ I would suggest removing this line. This study demonstrated that a ML model can identify those who did and did not complete flight training and provides insight on neural connectivity. It would be reasonable suggest a line how future research could employ these data to support those objectives.
